# Detecting Authoritarianism Efficiently: Psychometric Properties of the Screening Instrument *Authoritarianism – Ultra Short* (A-US) in a German Representative Sample

**DOI:** 10.3389/fpsyg.2020.533863

**Published:** 2020-11-24

**Authors:** Ayline Heller, Oliver Decker, Bjarne Schmalbach, Manfred Beutel, Jörg M. Fegert, Elmar Brähler, Markus Zenger

**Affiliations:** ^1^Department of Psychosomatic Medicine and Psychotherapy, University Medical Center, Johannes Gutenberg University Mainz, Mainz, Germany; ^2^Centre for the Study of Right-Wing Extremism and Democracy and Department of Medical Psychology and Medical Sociology, Leipzig University, Leipzig, Germany; ^3^Department of Medical Psychology and Medical Sociology, University Medical Center, Johannes Gutenberg University Mainz, Mainz, Germany; ^4^Department of Child and Adolescent Psychiatry/Psychotherapy, Ulm University, Ulm, Germany; ^5^Faculty of Applied Human Studies, Magdeburg-Stendal University of Applied Sciences, Stendal, Germany; ^6^Integrated Research and Treatment Center (IFB) Adiposity Diseases – Behavioral Medicine, Psychosomatic Medicine and Psychotherapy, University of Leipzig Medical Center, Leipzig, Germany

**Keywords:** authoritarianism, right-wing authoritarianism (RWA), screening instrument, screener, validation

## Abstract

With right-wing-extremist and -populist parties and movements on the rise throughout the world, the concept of authoritarianism has proven to be particularly valuable to explain the psychological underpinnings of these tendencies. Even though many scales to measure the different dimensions of authoritarianism exist, no short screening instrument has been tested and validated on a large scale so far. The present study examines the psychometric properties of the screening instrument *Authoritarianism – Ultrashort* (A-US) in three representative German samples (*n* = 2,524, *n* = 2,478, and *n* = 2,495). Using exploratory and confirmatory factor analysis, the A-US demonstrated acceptable internal consistency. Model fit was good and correlations with related constructs indicated convergent validity in both samples. Construct validity was demonstrated using the original version of the scale. The instrument proved to be invariant across sex, employment status, and education, but not across different age groups. Finally, the analyses showed that differences in the A-US are associated with sociodemographic variables. Potential causes and effects of these findings are discussed. Based on these results, the A-US proved to be a valuable and highly efficient tool to screen for authoritarian tendencies.

## Theoretical Background

The concept of authoritarianism first emerged with the rise of fascist movements in the 1930s, but it was not until the well-known *Studies in Prejudice* conducted by Adorno et al. (1950) that the concept was empirically tested. Based on their results, they developed a number of questionnaires to measure political attitudes of which the F-Scale became the best known (*F* as an abbreviation for fascism). It was conceptualized as an instrument to measure authoritarianism, defining it as a character trait consisting of nine distinct dimensions^1^. Following psychodynamic theory, they claimed that authoritarian character traits were formed mainly in early childhood and were largely dependent on the parent’s overly strict and harsh child rearing behavior. Even though the studies in general and the F-Scale in particular were criticized for various reasons, the idea of social and political attitudes being “ideologically organized along a single dimension that was a direct expression of personality” (Duckitt, 2015, p. 256) remained and so did the aim of finding adequate measurements.

In social science today, there is no homogeneous concept of authoritarianism. The phenomenon is still defined as a personality trait (e.g., Oesterreich, 2005) that mirrors social authoritarian dynamics (Decker, 2019). While the empirical findings evaluate the correlation between authoritarianism and prejudice, the concept was also adopted in social cognition. The double process model by Duckitt defines authoritarianism as a set of “social attitudinal or ideological expressions of basic social values or motivational goals that represent different, though related, strategies for attaining collective security at the expense of individual autonomy” (Duckitt and Bizumic, 2013). This definition focuses on the attitudinal and behavioral aspects as well as its effect on group processes rather than its etiology. Furthermore, it abandons a social theory approach to understand the social origins of authoritarian dynamics. In his notion of *right-wing authoritarianism* (RWA), Altemeyer (1981, 1988, 1996) reduces the original nine dimensions of the F-Scale to three, i.e., *authoritarian aggression*, *authoritarian submission*, and *authoritarian conventionalism*. Individuals with a high score in authoritarianism are thus expected to act aggressively toward an out-group or individuals showing socially deviant behavior, they prefer to follow the rule of a leader, and they are drawn to traditional values that are not to be scrutinized. In the present study, we rely on this definition to investigate the properties of our three-item, ultrashort screening scale *Authoritarianism -Ultra Short* (A-US). Using the well validated *Short Scale for Authoritarianism* (*Kurzzskala Autoritarismus*; KSA-3; Beierlein et al., 2014) as a basis, the A-US is aimed to measure the full range of authoritarianism, covering all three dimensions as defined by Altemeyer.

Authoritarianism can predict right-wing political attitudes as well as voting behavior (Decker and Brähler, 2006; Decker et al., 2016, 2018; Dunwoody and Plane, 2019). The concept shows overlap with the idea of conservatism as used, e.g., in a meta-analysis by Jost et al. (2003). Furthermore, when compared to the Big Five and Social Dominance Orientation, it has been shown to be one of the best predictors of generalized prejudices, especially when the out-group is perceived as threatening toward the social order and/or showing dissident behavior (Ekehammar et al., 2004; Duckitt and Sibley, 2007, 2009). It is thus associated with racism and sexism, as well as prejudice toward homosexuals and mentally disabled people (Ekehammar et al., 2004). Moreover, there is a correlation between acceptance of corporal punishment, violent educational methods and authoritarianism (Clemens et al., 2019). Authoritarianism fires the cycle of violence by approving child abuse and physical violence by parents and transmitting violence to the next generation (Clemens et al., 2020). Authoritarian attitudes are known to increase when the perceived threat on social and individual security is high (Asbrock et al., 2010; Asbrock and Fritsche, 2013; Dunwoody and Plane, 2019), making it an individual variable that is sensitive to changes to a given social situation. With anti-democratic parties and movements on the rise throughout the world and increasing violence against migrants and minorities, understanding and monitoring authoritarianism has become an issue of great political relevance. A reliable and efficient way of assessment lays the necessary foundation to work against these tendencies.

Altemeyer’s original scale, the *Right Wing Authoritarianism* (RWA), was designed to measure authoritarianism as a one-dimensional construct with three aspects. There is an ongoing debate about the dimensionality of authoritarianism though; Funke (2005) developed a three-dimensional, balanced scale that is among the most frequently used in German populations. Its items were criticized with regard to contents, involving questions about related concepts like prejudice, religiousness and conservatism.

The same holds true for the recently published *Very Short Authoritarianism Scale* (VSA) by Bizumic and Duckitt (2018). Their attempt to provide a short alternative to established measures builds on the well-validated, 18-item ACT-scale (Duckitt et al., 2010). It is made up of six items to capture the three aforementioned dimensions of authoritarianism using balanced two-item sets. While the ACT was developed to rid the RWA of its content overlap with criterion variables, the items operationalizing traditionalism or conventionalism are still likely to be culturally sensitive and show large overlap with religiousness^2^. While religiousness generally shows highs correlations with authoritarian attitudes, it is plausible that in certain subgroups or countries, there may be a different connection or no connection at all to authoritarian attitudes (e.g., in former socialist countries). In fact, Lee et al. (2018) found that the correlations of religiousness and political orientation largely vary across countries. Mixing the two constructs, authoritarianism and religiousness, in a single questionnaire may thus obscure the relationship between them. Moreover, with its six items, the VSA may still be unfit for some large-scale purposes.

Another widely used method of assessing authoritarianism efficiently applies questions regarding child-rearing values. The most prominent scale in this realm is the four-item *Authoritarian Child Rearing Values* (ACRV) and its adaptation, the ACRV-2, that has been used in the *American National Election Survey* (ANES). Participants are asked to choose between two item pairs of desirable qualities when raising a child, one representing authoritarian, the other non-authoritarian values. Even though correlations with the RWA and ACT can be considered acceptable, findings regarding reliability have been inconsistent (according to Bizumic and Duckitt, 2018, reported alphas range between 0.54 and 0.66 while they report an α = 0.71 themselves). Most importantly, it is doubtful that the ACRV-2 is capable of capturing all facets of authoritarianism as conceptualized by Altemeyer. Bizumic and Duckitt (2018) argue that while it might be used to operationalize authoritarian submission, authoritarian aggression may not be captured at all. Moreover, MacWilliams (2016) points out that there is an unsettled issue regarding cross-racial validity of the scale, as African–Americans might interpret the questions differently. Another substantial flaw regards the force-choice answering format. Opposition in meaning as well as equal social desirability of paired items in these formats is only assumed (Ray, 1990).

Beierlein et al. (2014) tried to eliminate some of these shortcomings by developing an unbalanced, nine-item short scale to measure authoritarianism in its three dimensions, the KSA-3. Unlike other short scales (e.g., Schmidt et al., 1995; Aichholzer and Zeglovits, 2015) its psychometric properties proved to be more than satisfactory. An ultrashort screening scale that covers the full spectrum of authoritarianism and is tested and validated using a representative sample has yet to be developed. It is needed in order to provide a more efficient way to screen for authoritarian tendencies within a society.

In the present study, we evaluate the three item, ultrashort version of the authoritarianism scale, based on the concept of Altemeyer (1988), and compare it to the original short scale by Beierlein et al. (2014). After an item analysis, an exploratory factor analysis (EFA) is used to analyze the dimensionality. It is then followed by a confirmatory factor analysis (CFA). As differences in authoritarianism and the support of right-wing extremist positions are often reported between certain groups (e.g., sex and age groups) and factors like employment status and educational background are used to explain mean differences, it is important to inspect measurement invariance as a prerequisite for comparing mean scores. To this end, measurement invariance is tested for these socio-demographic factors and their influence on mean and factor score is evaluated. Finally, construct validity is assessed using the original version of the scale and convergent validity is demonstrated using measures of right-wing attitudes, self-assessment of left/right positioning, as well as generalized and group specific prejudices.

## Materials and Methods

### Participants

The present study was part of a regular national representative survey of the general population of Germany. Two samples were analyzed using data collected in 2016 (Sample 1), 2017 (Sample 3), and 2018 (Sample 2), by an independent institute for opinion and social research (USUMA, Berlin). The criteria for inclusion were an age of ≥14 years and sufficient ability to understand the written German language. All adult participants provided their informed consent. In case of minors enrolled in the present study, informed consent was also obtained from the next of kin, caretakers, or guardians. After a sociodemographic interview, participants completed self-report questionnaires regarding political attitudes, physical and psychological symptoms in the presence (but without any interference) of the interviewer.

A random-route sampling procedure with 258 sample points revealed that 4,902 (Sample 1), 5,418 (Sample 2), and 5,160 (Sample 3) households should be contacted as part of the study. Of these, 4,830 households of Sample 1, 5,316 of Sample 2, and 5,093 of Sample 3 were eligible to participate (i.e., were not vacant or without individuals who met the inclusion criterion). The selection of the target persons within the households was carried out according to the Kish selection grid. In total, there were 2,524 participants in Sample 1, 2,516 in Sample 2, and 2,531 in Sample 3 (participation rate 52.7, 47.5, and 49.7% respectively). Due to the shortness of the scale, only participants that completed all three items of the A-US were included, leading to an exclusion of *n* = 79 (Sample 1) and *n* = 38 (Sample 2). As Sample 3 was used for construct validation, all participants with missing values in the nine-item version of the scale were excluded (*n* = 36). Thus, the final samples consisted of 2,465 (Sample 1), 2,478 (Sample 2) and 2,495 subjects (Sample 3). Sociodemographic characteristics of the study samples are presented in Table 1. While the three samples did not show notable differences, when comparing the sex and age groups to data provided by the Federal Statistical Office of Germany (2019), a slight overrepresentation of female participants as well as an underrepresentation of younger age groups could be observed. As these were minor deviations, the data can be assumed to be representative of the German population.

**TABLE 1:** Sample description based on A-US scores.

	**Sample 1**	**Sample 2**	**Sample 3**	
	***N* = 2,524**	***N* = 2,478**	***N* = 2,495**	
**Sex**	***N (%)***	***N (%)***	***N (%)***	**% in population**

Female	1,344 (54.5)	1,350 (54.5)	1,382 (55,4)	50.7
Male	1,121 (45.5)	1,128 (45.5)	1,113 (44.6)	49.3

**Age**	***M (SD)***	***M (SD)***	***M (SD)***	

Age, years	48.93 (18.17)	48.02 (17.48)	48.58 (17.92)	
Age range	14 – 99	14 – 91	14 – 93	

**Age groups**	***N (%)***	***N (%)***	***N (%)***	

14–29 years	441 (17.9)	453 (18.3)	476 (19.1)	20.4
30–39 years	360 (14.6)	376 (15.2)	358 (14.3)	14.4
40–49 years	433 (17.6)	437 (17.6)	392 (15.7)	14.8
50–59 years	482 (19.6)	522 (21.1)	516 (20.7)	18.5
60–69 years	392 (15.9)	416 (16.8)	423 (17.0)	13.9
≥70 years	357 (14.5)	274 (11.1)	330 (13.2)	18.0
**Relationship status**				
Married/living together	1,018 (41.3)	1,058 (42.7)	1,052 (42.2)	
Married/separated	53 (2.2)	70 (2.8)	64 (2.6)	
Committed relationship	262 (10.6)	358 (14.4)	252 (10.1)	
Unmarried	590 (23.9)	515 (20.8)	637 (25.5)	
Divorced	298 (12.1)	287 (11.6)	273 (10.9)	
Widowed	237 (9.6)	183 (7.4)	206 (8.3)	
Missing	7 (0.3)	7 (0.3)	11 (0.4)	
**Education**				
≤8 years	802 (32.5)	859 (34.6)	774 (31.0)	
9 – 11 years	1,059 (43.0)	1,050 (42.3)	1,123 (45.0)	
≥12 years	529 (21.4)	504 (20.3)	530 (21.3)	
School student	73 (3.0)	60 (2.4)	60 (2.4)	
Missing	2 (0.1)	5 (0.2)	8 (0.3)	
**Employment status**				
Education/training	208 (8.4)	185 (7.4)	205 (8.2)	
Working	1,294 (52.5)	1,381 (55.7)	1,334 (53.5)	
Unemployed/working < 15h per week	222 (9.1)	240 (9.7)	233 (9.3)	
Homemaker	85 (3.4)	76 (3.1)	78 (3.1)	
Retired	636 (25.8)	572 (23.1)	631 (25.3)	
Missing	20 (0.8)	24 (1.0)	14 (0.6)	

*A-US, authoritarianism – ultra short; % in pop., population values according to the Federal Statistical Office of Germany (2019).*

The study was approved by the Ethical Committee of the Leipzig University (Az: 452-15-21122015 for Sample 1, Az: 132/18-ek for Sample 2 and Az: 418/17-ek for Sample 3).

### Measures

For the present study, we used a three-item version of the *Short Scale for Authoritarianism* (*Kurzzskala Autoritarismus*; KSA-3; Beierlein et al., 2014) that is designed to measure authoritarianism on a five-point scale, with 1 indicating strong opposition and 5 indicating strong agreement. The original scale consists of nine items on three dimensions (i.e., aggression, submission, and conventionalism). The items with the highest factor loadings on each dimension were selected for the ultrashort, three-item version, the *Authoritarianism – Ultra Short* (A-US). This type of item selection insured that the three original dimensions were best represented in the short scale. An overall score was computed by adding the individual scores of each of the three selected items of the ultrashort scale. Original item wording as well as an English translation are provided in Table A1 in the Appendix. Additionally, for construct validation, a shortened six-item score of the original scale was calculated by adding up the scores of the remaining items not selected for the A-US.

The *Leipzig Scale on Right-Wing Extremist Attitudes* (*Fragebogen zur Rechtsextremen Einstellung*; FR-LF; Decker et al., 2013) assesses right-wing attitudes using six dimensions. Each dimension consists of three items that are to be rated on a five-point scale ranging from 1 = *I fully disagree* to 5 = *I fully agree.* Decker et al. (2013) found the questionnaire showed a very good internal consistency of α = 0.94. For this study, the total score was used by adding up all item scores.

Political orientation was measured using a single-item left-right-self assessment scale (“*Thinking about your own political views, how would you rate them on the following scale?”*) ranging from 1 = *left* to 10 = *right*.

Generalized and group specific prejudices were analyzed using parts of the questionnaire developed by the research group around Heitmeyer (2012). It assesses several forms of group-related hostility (*Gruppenbezogene Menschenfeindlichkeit*; GMF) on a four-point scale ranging from 1 = *I fully agree* to 4 = *I fully disagree*. To make the results more accessible, all necessary items were poled so that high scores indicated high values of GMF. In the present study, we took items measuring prejudices against Muslims (two items; ω_1_ = 0.84; ω_2_ = 0.83), and Sinti and Roma (three items; ω_1_ = 0.90; ω_2_ = 0.91) from both Samples. Items regarding homophobic attitudes (three items, one inverted; ω_1_ = 0.83) as well as sexism (two items, ω_1_ = 0.86) were included using additional data from Sample 1. An overall score to account for generalized prejudices was also calculated by adding all used items (ten in Sample 2 and five in Sample 2; ω_1_ = 0.86; ω_2_ = 0.89)^3^.

### Statistical Analyses

On Sample 1, an EFA was conducted to determine the number of factors of the A-US. We then used Sample 2 to confirm the findings using confirmatory factor analysis (CFA). Both subsamples did not differ significantly with regard to A-US mean scores, sex, and age (see Tables 1, 2).

**TABLE 2:** Descriptive statistics and factor loadings of the A-US items in Sample 1 and Sample 2.

**Item/Scale**	**Sample**	***M* (*SD*)**	**Range**	**Skewness**	**Kurtosis**	***P***	***r*_*it*_**
Item 1: Troublemakers should clearly feel the effects of the fact that they are unwanted in the society.	1 2	3.88 (1.18) 3.82 (1.16)	1 – 5	−0.87 −0.70	−0.13 −0.45	0.72 0.71	0.41 0.44
Item 2: People should leave important decisions to those in charge/the leaders.	1 2	2.67 (1.14) 2.68 (1.11)	1 – 5	0.23 0.12	−0.70 −0.73	0.42 0.42	0.50 0.47
Item 3: Established conducts should not be questioned.	1 2	3.16 (1.15) 3.14 (1.13)	1 – 5	−0.14 −0.15	−0.68 −0.73	0.54 0.54	0.55 0.57

A-US mean score	1 2	3.24 (0.90) 3.21 (0.88)	1 – 5	−0.26 −0.19	−0.27 −0.44		

*A-US, authoritarianism – ultra short; P, difficulty index; *r*_*it*_, corrected item-total correlation.*

For the EFA, principal axis factoring was applied using SPSS. A total of three different indicators were used to identify the factor structure of the A-US: Kaiser Guttman criterion, scree-plot, and Horn’s parallel analysis (PA; Horn, 1965). PA focuses on extracting Eigenvalues from random data sets that have the same number of cases and variables as the original raw data. This procedure is based on the idea that factors of real data should have larger Eigenvalues that those extracted from random data. Consequently, only those factors were retained in the real data that showed Eigenvalues greater than those of the random data (O’Connor, 2000). The parallel analysis engine provided by Patil et al. (2017) was used to create random data. The method was based on PCA factor extraction and used 95th percentile of the Eigenvalues as a threshold instead of the mean to avoid overextraction of factors.

Additionally, a CFA was conducted on Sample 2 to confirm the factorial structure of the A-US. To this end, we used R and the packages *lavaan* and *semTools* (Rosseel, 2012; semTools Contributors, 2016) and each model was estimated with the robust maximum likelihood method approach (Satorra and Bentler, 2001). Due to the shortness of the A-US with only three items, model fit indices could not be calculated, as a model with three indicators of a latent variable is just-identified. As we did not want to impose additional constraints to the model, only factor loadings and a measure of internal consistency, McDonald’s (1999)ω (Trizano-Hermosilla and Alvarado, 2016), are reported.

Additional analyses were conducted using Sample 1 and Sample 2 to test the invariance of the model across sex and age as well as education and employment status using multi-group CFA in R (Meredith, 1993). After testing the factorial structure in each subgroup, measurement invariance was tested in three steps using the configural model first (without constraints), followed by a metric invariant model (with factor loadings constrained to be equal across groups), a scalar invariant model (with factor loadings and item intercepts simultaneously constrained to be equal across groups), and a strict invariant model (with factor loadings, item intercepts, and residuals constrained to be equal across groups). Due to the hierarchy of these nested and increasingly restrictive models, they could then be compared. Due to the large sample size, the χ^2^ significance-test was capable of detecting even the smallest model differences (e.g., Putnick and Bornstein, 2016). A non-significant χ^2^ test result could thus be seen as a very strong indicator that invariance holds. For the cases of significant χ^2^ results we reported differences Δ *CFI* and Δ *gamma Hat* (*GH*, Steiger, 1989) as alternative measures. Values equal to or smaller than 0.01 indicated the invariance of the model (Cheung and Rensvold, 2002; Milfont and Fischer, 2010). Whenever full scalar invariance could not be assumed, partial invariance was tested by consecutively constraining only two of the three item intercepts to be equal across groups while one was estimated freely. Even though stepwise selection processes like this have been heavily criticized (see Marsh et al., 2018), Gregorich (2006) argues that partial invariance allows for valid comparisons in mean scores as long as two loadings and intercepts are constrained to be equal across groups. Nevertheless, the assumption of partial invariance should always be considered inferior to full scalar invariance.

The combined sample was then used to identify possible influences of sociodemographic factors on A-US scores within the SEM framework. For this, latent means were fixed to be equal across groups and model fit was analyzed once again. A significant decline in model fit compared to the strict invariance model was seen as an indicator that differences were present. Latent means were then compared in the strict invariance model between the groups. Finally, *R*^2^ was calculated to show the extent of the differences found by comparing between-group-variance in intercepts and latent means to the pooled total variance.

Finally, Sample 3 was used for construct validation calculating Pearson correlation with a reduced version of the original scale containing only those six items not included in the A-US. Correlations were analyzed in all relevant subgroups. Furthermore, Pearson correlations were used to explore convergent validity of the A-US with related constructs, i.e., right-wing extremist attitudes, left-right-self-assessment, and different measures of prejudice.

## Results

### Descriptive Statistics

Descriptive statistics for each item were reported separately for Sample 1 and Sample 2 in Table 2. While skewness and kurtosis lay within the commonly agreed upon cut-offs of <2 (Pituch and Stevens, 2016), histograms showed clear deviations from normality on an item level, especially in item 1 (data not presented). Due to these findings, we assumed a non-normal distribution of data on the item level but not for the scale as a whole. We thus used robust estimation and fit indices for the EFA and CFA whenever available (Brosseau-Liard et al., 2012; Brosseau-Liard and Savalei, 2014), as these methods are based on an item level, but parametric measures when scale scores were involved (e.g., for mean comparisons and correlations). Difficulty indices for the A-US items ranged between 0.42 (Item 2) and 0.72 (Item 1), indicating a medium to low item difficulty within the accepted range of 0.20 to 0.80 (Moosbrugger and Kelava, 2012). Furthermore, the corrected item-total correlations all scored above the cut-off of 0.40 (Moosbrugger and Kelava, 2012). No notable differences between the two samples were observed.

### EFA, CFA, and Reliability

Results of the EFA indicated a unidimensional one-factor solution using the Kaiser Guttman criterion with an Eigenvalue of 1.29, accounting for 42.92% of the variance. Factor loadings for the three items were 0.50, 0.65, and 0.78 respectively. The visual evaluation method of the scree-plot indicated one factor as well (data not shown). Additionally, PA (Horn, 1965) indicated a unidimensional scale structure with only the Eigenvalue of the first factor exceeding the value of the Random Data (see Table 3).

**TABLE 3:** Results of parallel analysis using Sample 1.

**Factors**	**PA Eigenvalues**	
	**Raw data**	Random data*
1	1.824	1.032
2	0.695	1.000
3	0.481	0.968

PA, parallel analysis. *Eigenvalues corresponding to the 95th percentile of the distribution of random data eigenvalues, which are based on 1,000 random data sets.

According to the results of the EFA, the unidimensional model was tested by CFA in Sample 2. As the model was just identified, fit indices could not be calculated. Factor loadings were 0.53, 0.59, and 0.81 for the three items. Reliability analysis led to an ω*_1_* = 0.68 in Sample 1, ω*_2_* = 0.69 in Sample 2 and ω*_3_* = 0.71 in Sample 3.

### Measurement Invariance

Measurement invariance of the A-US was tested regarding the following groups using both Sample 1 and Sample 2: sex, age, employment status, and education. Results are shown in Table 4.

**TABLE 4:** Tests for invariance across sex, age groups, employment status and education.

	**χ^2^*(df)***	**Δχ^2^ (Δ *df*)**	***P***	***CFI***	**Δ *CFI***	***GH***	**Δ *GH***
**Sex**							
Configural model	0 (0)			1.000		1.000	
Metric model	1.641 (3)	1.641 (3)	0.650	1.000	<0.001	1.000	<0.001
Scalar model	5.472 (5)	3.831 (2)	0.147	1.000	<0.001	1.000	<0.001
Strict model	6.152 (8)	2.321 (3)	0.509	1.000	<0.001	1.000	<0.001
Latent means	6.788 (9)	0.636 (1)	0.425	1.000	<0.001	1.000	<0.001
**Age groups**							
Configural model	0 (0)			1.000		1.000	
Metric model	20.084 (15)	20.084 (15)	0.169	0.998	0.002	0.999	0.001
Scalar model	62.084 (25)	42.000 (10)	<0.001	0.984	0.014	0.995	0.004
Partial scalar invariance: item 1	54.523 (20)	34.439 (5)	<0.001	0.985	0.015	0.995	0.004
Partial scalar invariance: item 2	30.671 (20)	10.587 (5)	0.060	0.995	0.003	0.999	0.001
Partial scalar invariance: item 3	31.838 (20)	11.754 (5)	0.038	0.995	0.003	0.991	0.008
Strict model^*a*^	94.335 (35)	63.664 (15)	<0.001	0.975	0.020	0.971	0.020
Latent means^*a*^	259.485 (40)	165.150 (5)	<0.001	0.909	0.066	0.971	<0.001
**Employment status**							
Configural model	0 (0)			1.000		1.000	
Metric model	10.175 (12)	10.175 (12)	0.601	1.000	<0.001	1.000	<0.001
Scalar model	38.312 (20)	28.137 (8)	<0.001	0.991	0.009	0.998	0.002
Strict model	56.817 (32)	18.505 (12)	0.101	0.989	0.002	0.996	0.002
Latent means	193.820 (36)	136.003 (4)	<0.001	0.932	0.057	0.979	0.017
**Education**							
Configural model	0 (0)			1.000		1.000	
Metric model	8.087 (6)	8.087 (6)	0.232	0.999	0.001	1.000	<0.001
Scalar model	17.518 (10)	9.432 (4)	0.051	0.996	0.003	0.998	0.002
Strict model	45.312 (16)	27.794 (6)	<0.001	0.986	0.010	0.995	0.003
Latent means	284.604 (18)	239.292 (2)	<0.001	0.879	0.107	0.964	0.031

*df, degrees of freedom; CFI, comparative-fit-index; GH, gamma Hat. ^*a*^Intercepts of item 2 vary between groups.*

Due to the non-significant χ^2^-test as well as Δ*CFI* and Δ*GH* < 0.01, the A-US could be assumed strictly invariant across sex. The results for different age groups were less clear. While both the configural and metric model proved to be sufficient using the relevant indices, complete scalar invariance could not be assumed. Therefore, all variants of partial invariance were explored by consecutively freeing the intercepts of each item across the groups while fixing the other two item intercepts to be equal across groups. While there was no significant effect of allowing for variation of intercepts across groups for item 1, freely estimating the intercepts across groups of either item 2 or item 3 lead to partial invariance. The intercepts showed a linear age trend with older age groups showing higher values in the intercepts (see Table 5). As the free estimation of intercepts across groups for item 2 lead to the best model fit, we then tested for strict invariance including this constraint. All relevant indices showed a significant decline in model fit. Therefore, strict invariance could not be assumed.

**TABLE 5:** Unstandardized item intercepts and latent means across groups.

	***N***	**Latent mean**	**Intercept item 1**	**Intercept item 2**	**Intercept item 3**
**Sex**					
Men	2,249	0	3.819	2.679	3.135
Women	2,694	0.027	3.880	2.668	3.160
**Age**					
18–29 years	894	0	3.689	2.597	2.894
30–39 years	736	0.055	3.780	2.614	2.951
40–49 years	870	0.161	3.810	2.641	3.064
50–59 years	1,004	0.281	3.909	2.655	3.199
60–69 years	808	0.365	3.926	2.668	3.292
≥70 years	631	0.705	4.030	2.927	3.591
**Employment status**					
Education/training	393	−0.265	3.659	2.575	2.791
Working	2,675	0	3.808	2.640	3.079
Unemployed/working < 15 h per week	462	−0.064	3.844	2.552	3.017
House wife/man	161	0.063	3.888	2.652	3.137
Retired	1,208	0.392	4.002	2.821	3.478
**Education**					
≤ 8 years	2109	0.293	4.037	2.842	3.409
9 – 11 years	1033	0	3.871	2.666	3.139
≥12 years	1661	−0.435	3.527	2.411	2.767

Regarding employment status and education^4^, strict invariance could be assumed based on the analyses. Even though employment status showed significant χ^2^-test results for the scalar model, both Δ*CFI* and Δ*GH* remained below the cut-off of 0.01. As χ^2^-tests are capable of detecting even the slightest effects in larger samples, *CFI* and *GH* are the more reliable indices in this case (Putnick and Bornstein, 2016). Intercepts as well as latent means for the different employment statuses can be found in Table 5. Compared to the group working in full time, the group of people in training as well as those working less than 15 h per week showed lower A-US intercepts and latent mean scores, while homemakers as well as retired persons showed higher intercepts and latent means scores. The analysis of measurement invariance for education status lead to similar results. The strict model showed a significant χ^2^-test result and Δ*CFI* fell on the cut-off of 0.01. As Δ*GH* was still far below the cut-off score, strict invariance could be assumed. A linear trend regarding the years of education was found in the intercepts as well as latent mean scores (see Table 5).

### Influence of Sociodemographic Parameters

As Table 4 shows, a significant decline in model fit could be observed in all sociodemographic parameters except sex when fixing latent mean scores across groups. Therefore, age group, education and employment status all have an influence on A-US mean scores. No mean differences were found between men and women.

Even though full scalar invariance was not given in the case of age groups, meaningful comparisons of latent means are still possible (Gregorich, 2006). The results revealed a linear trend with older participants showing higher authoritarianism in a descriptive manner (see Table 5). Differences in latent means between the age groups accounted for 4.53% of the total variance. Regarding employment status, group membership accounted for 3.95% of the total variance. The group of retired people showed the highest A-US latent mean scores while those in education or training showed the lowest. This may at least partially be explained by an overlap with the reported age effect. Interestingly, the group of unemployed and working < 15 h per week showed lower A-US scores than the group of those working full time. Finally, education had the largest influence on authoritarianism, explaining 9.36% of the total variance. Once again, a linear trend could be observed. Participants with a higher level of education showed lower scores in authoritarianism.

Overall, a high influence of most of the sociodemographic parameters was revealed. This goes in line with previous studies on authoritarianism and right-wing extremist attitudes and needs to be taken into consideration for future research on the topic.

### Construct Validity

To analyze construct validity, Pearson correlation with a reduced, six-item version of the scale was assessed, to account for the item overlap. The correlation was very high (*r* = 0.879). Testing different age groups, educational backgrounds and employment statuses did not reduce the correlations substantially. As shown in Table 6, correlations between the three items of the A-US and the remaining six items of the original scale ranged between 0.862 and 0.923 in all groups. This can be regarded as very strong evidence that the proposed ultra-short scale indeed measures the same construct as the original scale.

**TABLE 6:** Correlations of the A-US with a reduced version of the original scale.

	**KSA-reduced**
**Sex**	
Men	0.873
Women	0.883
**Age**	
18–29 years	0.873
30–39 years	0.880
40–49 years	0.864
50–59 years	0.878
60–69 years	0.884
≥ 70 years	0.867
**Employment status**	
Education/training	0.838
Working	0.877
Unemployed/working < 15 h per week	0.907
House wife/man	0.923
Retired	0.869
**Education**	
≤8 years	0.867
9 – 11 years	0.878
≥12 years	0.862

*A-US, authoritarianism – ultra short; KSA-reduced, six-item version of the original short scale, without items of A-US.*

### Convergent Validity

To analyze the validity of the scale, correlations with adjacent constructs were calculated (see Table 7). We expected positive correlations with right-wing attitudes, left-right self-assessment as well as generalized and group specific prejudices. All of these were found to be highly significant in both samples, showing small to medium effect sizes ranging from *r* = 0.16 in the left-right-self-assessment in Sample 1 to *r* = 0.50 in right-wing extremism in Sample 2 (Cohen, 1988).

**TABLE 7:** Correlations between A-US and related psychological measures.

	**Sample 1**	**Sample 2**
	***r (N)***	***r (N)***
Right-wing extremisms	0.39** (2,259)	0.50** (2,346)
Left-right-self-assessment	0.15** (2,404)	0.20** (2,429)
Prejudice	0.34** (2,325)	0.31** (2,409)
against Muslims	0.22** (2,437)	0.29** (2,456)
against Sinti and Roma	0.27** (2,388)	0.27** (2,418)
against Homosexuals	0.26** (2,402)	–
Sexism	0.22** (2,428)	–

A-US, authoritarianism – ultra short. **p < 0.01. r, Pearson correlation coefficient.

## Discussion and Limitations

The aim of the present study was to examine the psychometric properties of the ultra-short screening instrument for authoritarianism A-US. A short scale to monitor authoritarian tendencies in large samples is crucial to understand and predict changes in the social climate.

The A-US showed good to excellent psychometric properties. Non-normal distribution of data was assumed on an item level but not for the scale as a whole. This was taken into consideration by using robust estimation and fit indices for the tests of measurement invariance. Factorial validity was assessed by EFA and CFA. Both methods confirmed the one-dimensionality of the A-US with factor loadings ranging between 0.50 (Item 1 in the EFA) and 0.81 (Item 3 in the CFA). Moreover, internal consistency (McDonald’s ω) ranged between 0.68 and 0.71 in all samples. Taking into consideration the shortness and purpose of the three-item instrument, this can be evaluated as satisfying. It is also comparable to the aforementioned VSA. As the A-US is intended for group statistics rather than individual assessment, efficiency may be ranked higher than internal consistency (Ziegler et al., 2014).

Tests of measurement invariance revealed strict invariance of the A-US across sex, employment status and education. This is an important statistical prerequisite that allows for meaningful observed mean comparisons between these groups (Gregorich, 2006). As for the age groups, partial scalar invariance was given when freely estimating the intercepts between groups for either item 2 or item 3. Even though results should be considered with some care as the stepwise procedure of testing for partial invariance has been criticized as possibly leading to idiosyncratic results (Marsh et al., 2018), this allows for meaningful comparisons of intercepts and latent mean scores across groups. Strict invariance could not be assumed across age groups, so observed mean scores should not be compared at all.

Some important mean differences were found in relation to sociodemographic parameters. A linear age trend was observed in all item intercepts as well as latent mean scores. In the case of item 3, age group membership accounted for 4.52% of the total variance of authoritarianism. As this item represents the dimension *authoritarian conventionalism* of the original scale, the results imply that older age groups tend to hold more conservative and traditional attitudes, while also showing more authoritarianism in general. With the data at hand, it is not possible to clarify whether this is a general age trend, due to e.g., changes in personality and cognition associated with older age (Cornelis et al., 2009) or whether the differences are due to birth cohort. As this scale was tested in a German population, the effect may be viewed as a relic of the Nazi-era and the resulting scores might be caused by a more authoritarian environment the older age groups were raised in. International samples should be used to clarify this effect and further analyses should try to separate age effects from birth cohort effects. Employment status accounted for 3.95% of the total variance using a comparison of latent mean scores. As the group of retired people showed the highest scores in authoritarianism and the group of people in training and education showed the lowest, an overlap with age effect is likely. Furthermore, the largest effects were found regarding the educational background where higher authoritarianism scores were associated with lower educational levels. Group membership explained 9.36% of the variance in latent mean scores. This finding once again proves that higher education may serve as a buffer for authoritarian and right-wing extremist attitudes (e.g., Rippl, 2002; Zick et al., 2011; Decker et al., 2018).

Construct validity was assessed using Pearson correlations with the long version of the scale. The correlations with the reduced version excluding the three items of the short scale were very high (*r* = 0.879). Convergent validity was demonstrated by small to medium correlations to other instruments measuring related constructs. The relatively low correlation with left-right-self assessment may be explained mainly by the fact that it is a single-item instrument. In the validation study by Beierlein et al. (2014), two out of three subdimensions showed similar correlations of *r* = 0.22 (*authoritarian submission*) and *r* = 0.21 (*conventionalism*) with political left-right-self assessment compared to the A-US. Correlations of these two dimensions with prejudices against homosexuals and migrants ranged between *r* = 0.27 and *r* = 0.34, and were thus comparable to those of the A-US as well. The dimension *authoritarian aggression* of the KSA-3 generally reached higher correlations with prejudices and political self-assessment than the A-US. Taking into consideration the relatively low factor loading of the first item of the A-US taken from that dimension, this may be seen as an indicator, that *authoritarian submission* and *conventionalism* are better captured by the A-US than *authoritarian aggression*. Some other scales assessing authoritarianism have been criticized for showing an overlap in item-contents and -wording with the parameters they want to explain, leading to an increase in estimated correlations. As this short scale uses rather broadly worded items, little to no overlap is to be expected^5^. The overall correlational pattern of this short scale can thus be viewed as a strong indicator of validity, even though the effect sizes only indicate small to medium effects.

Limitations include the one-dimensionality of the scale that does not fully reflect the complexity of the construct (Funke, 2005, see above). This may be intensified by the fact that the A-US only uses three items to capture a construct that was originally made up of three dimensions. In most studies, authoritarianism is treated as a unitary parameter though (Beierlein et al., 2014), suggesting that this simplification may not result in grave impairments of the quality of the scale. Another potential problem regards the reliability of short scales, as they, once again, may not capture the full complexity of the constructs they are trying to measure. The A-US showed high internal consistency and factor loadings as well as high correlations with the original scale and related constructs. Considering the anticipated use of the scale, it thus demonstrates adequate reliability and validity. Comparisons with older data and other authoritarianism scales could be used to further improve construct validity. Unfortunately, no data is available yet to cover an adequate time frame needed for an analysis of the stability of the A-US over time. Still, the results of this study indicate that the A-US is able to capture authoritarianism in most relevant research and screening contexts. Finally, the one-sided answering format may lead to acquiescence (MacWilliams, 2016). According to Podsakoff et al. (2003), acquiescence reflects “the propensity for respondents to agree (or disagree) with questionnaire items independent of their content” (p. 887). This may especially be a problem when it comes to authoritarianism, a concept already closely linked to conformity, i.e., the tendency to agree. Balanced short-scales, like the VSA, may account for this by controlling for the direction of item wording. In this respect, they may be better at differentiating between high scoring “authoritarians” and mere “conformists” that show a tendency to agree. With its six items, the VSA may still be too long for some research purposes though. In an ultra-short scale like the A-US, reversed item wording in one of only three items may lead to additional problems, a possible distortion in item meaning being the most important of them (cf. Rokeach, 1967). In scale construction, we relied on the well-validated KSA-3 by Beierlein et al. (2014) that is capable of capturing the three dimension of authoritarianism as conceptualized by Altemeyer. In the three-item version we proposed, our focus was to adequately reflect the whole spectrum of authoritarianism rather than creating a balanced scale based on item wording. To further evaluate and address the problem of response biases, future research should aim to translate and validate both the ACRV-2 and VSA short scales for the use in German populations in order to allow for a more meaningful comparison with the A-US. These possible impairments should be kept in mind when using this short scale. As this instrument is supposed to serve only as a very short screener for authoritarian tendencies in large groups, rather than a measure for individual assessment, most of these shortcomings seem negligible (Ziegler et al., 2014).

Due to these deficits, more elaborated scales should be preferred for complex analyses on the genesis and effects of authoritarianism. The influence of sociodemographic factors on authoritarianism should be taken into consideration at all times and the issue of possible response biases should be addressed in future research. Based on the results altogether, the A-US proved to be a valid and reliable screening tool for large-scale assessment and monitoring of authoritarian tendencies.

## Data Availability Statement

The datasets generated for this study are available on request to the corresponding author.

## Ethics Statement

The studies involving human participants were reviewed and approved by Ethics committee of the Faculty of Medicine at the University of Leipzig (Az: 452-15-21122015 for Sample 1 and Az: 132/18-ek for Sample 2). Written informed consent to participate in this study was provided by the participants’ legal guardian/next of kin.

## Author Contributions

MZ, OD, and EB contributed to the conception and design of the study. EB, OD, and JF organized the database. AH, MZ, and BS performed the statistical analyses. AH and MZ wrote the first draft of the manuscript. All the authors were involved in the revision of the first and subsequent drafts, read and approved the submitted version.

## Conflict of Interest

The authors declare that the research was conducted in the absence of any commercial or financial relationships that could be construed as a potential conflict of interest. The reviewer MM declared a shared affiliation, with no collaboration, with one of the authors, JF, to the handling editor at the time of the review.

## Footnotes

1 *Support for conventional values*, *authoritarian submission*, *authoritarian aggression*, *stereotypy and rigidity*, *toughness and power*, *cynicism*, as well as *anti-introception*, *projectivity*, and *sexual inhibition* on a psychodynamic level (Saunders and Ngo, 2017).

2 “God’s laws about abortion, pornography, and marriage must be strictly followed before it is too late.” and “There is nothing wrong with premarital sexual intercourse.”

3 Only participants answering all items were included in the calculation of ω (see Table 7 for sample sizes).

4 For the analyses on education, only those participants who had already finished their education were included.

5 The only potential overlap might be found between item 2 and the subscale *Affinity toward Authoritarian Regimes* of the FR-LF.

## Appendix

**TABLE A1:** A-US items in German and English.

	**German original**	**English translation**
Item 1	Unruhestifter sollten deutlich zu sp ren bekommen, dass sie in der Gesellschaft unerw nscht sind.	Troublemakers should clearly feel the effects of the fact that they are unwanted in the society.
Item 2	Menschen sollten wichtige Entscheidungen in der Gesellschaft F hrungspersonen berlassen.	People should leave important decisions to those in charge/the leaders.
Item 3	Bew hrte Verhaltensweisen sollten nicht in Frage gestellt werden.	Established conducts should not be questioned.

## References

[B1] Adorno T. W. Frenkel-Brunswik E. Levinson D. J. Sanford R. N. (1950). *The Authoritarian Personality: Studies in Prejudice.* Available online at: http://www.ajcarchives.org/main.php?GroupingId=6490 (Accessed November 29, 2019).

[B2] Aichholzer J. Zeglovits E. (2015). “Balancierte Kurzskala autoritärer Einstellungen (B-RWA-6) [Balanced short scale of authoritarian attitudes],” in *Zusammenstellung Sozial-wissenschaftlicher Items und Skalen*, eds Danner D. Glöckner-Rist A. (Mannheim: GESIS). 10.6102/zis239

[B3] Altemeyer B. (1981). *Right-wing Authoritarianism.* Winnipeg: University of Manitoba Press.

[B4] Altemeyer B. (1988). *Enemies of Freedom: Understanding Right-wing Authoritarianism.* San Francisco, CA: Jossey-Bass.

[B5] Altemeyer B. (1996). *The Authoritarian Specter.* Cambridge, MA: Harvard University Press.

[B6] Asbrock F. Fritsche I. (2013). Authoritarian reactions to terrorist threat: who is being threatened, the Me or the We? *Int. J. Psychol.* 48 35–49. 10.1080/00207594.2012.695075 23390971

[B7] Asbrock F. Sibley C. G. Duckitt J. (2010). Right-wing authoritarianism and social dominance orientation and the dimensions of generalized prejudice: a longitudinal test. *Eur. J. Pers.* 24 324–340. 10.1002/per.746

[B8] Beierlein C. Asbrock F. Kauff M. Schmidt P. (2014). *Die Kurzskala Autoritarismus (KSA-3): Ein ökonomisches Messinstrument zur Erfassung dreier Subdimensionen autoritärer Einstellungen [The Short Scale for Authoritarianism (KSA-3): An Efficient Instrument to Measure Three Subdimensions of Authoritarian Attitudes].* Mannheim: GESIS – Leibniz Institut für Sozialwissenschaften.

[B9] Bizumic B. Duckitt J. (2018). Investigating right wing authoritarianism with a very short authoritarianism scale. *J. Soc. Political Psychol.* 6 129–150. 10.5964/jspp.v6i1.835

[B10] Brosseau-Liard P. E. Savalei V. (2014). Adjusting incremental fit indices for nonnormality. *Multiv. Behav. Res.* 49 460–470. 10.1080/00273171.2014.933697 26732359

[B11] Brosseau-Liard P. E. Savalei V. Li L. (2012). An investigation of the sample performance of two nonnormality corrections for RMSEA. *Multiv. Behav. Res.* 47 904–930. 10.1080/00273171.2012.715252 26735008

[B12] Cheung G. W. Rensvold R. B. (2002). Evaluating goodness-of-fit indexes for testing measurement invariance. *Struct. Equ. Model.* 9 233–255. 10.1207/s15328007sem0902_5

[B13] Clemens V. Decker O. Plener P. L. Brähler E. Fegert J. M. (2019). Authoritarianism becomes respectable in Germany: a risk factor for condoning physical violence toward children? *Zeitschrift Kinder Jugendpsychiatrie Psychother.* 47 1–13. 10.1024/1422-4917/a000684 31414925

[B14] Clemens V. Decker O. Plener P. L. Witt A. Sachser C. Brähler E. (2020). Authoritarianism and the transgenerational transmission of corporal punishment. *Child AbuseNeglect* 106:104537. 10.1016/j.chiabu.2020.104537 32422465

[B15] Cohen J. (1988). *Statistical Power Analysis for the Behavioral Sciences.* Hillsdale, MI: Erlbaum.

[B16] Cornelis I. Hiel A. V. Roets A. Kossowska M. (2009). Age differences in conservatism: evidence on the mediating effects of personality and cognitive style. *J. Pers.* 77 51–88. 10.1111/j.1467-6494.2008.00538.x 19076995

[B17] Decker O. (2019). Secondary authoritarianism – the economy and right-wing extremist attitudes in contemporary Germany. *J. Psychosoc. Stud.* 12 203–213. 10.1332/147867319X15608718111032

[B18] Decker O. Brähler E. (2006). *Vom Rand zur Mitte: Rechtsextreme Einstellungen und ihre Einflussfaktoren in Deutschland [From the Margin to the Middle: Extreme Right-wing Attitudes and Influencing Factors in Germany].* Berlin: Friedrich-Ebert-Stiftung.

[B19] Decker O. Hinz A. Geißler N. Brähler E. (2013). “Fragebogen zur rechtsextremen Einstellung - Leipziger Form (FR-LF) [Questionnaire for right-wing extremist attitudes – Leipzig form],” in *Rechtsextremismus der Mitte: Eine Sozialpsychologische Gegenwartsdiagnose*, eds Decker O. Kiess J. Brähler E. (Gießen: Psychosozial-Verlag), 197–212. 10.30820/9783837966367-197

[B20] Decker O. Kiess J. Brähler E. (2016). *Die enthemmte Mitte: Autoritäre und rechtsextreme Einstellung in Deutschland 2016 [The Disinhibited Middle-class: Authoritarian and Extreme Right-wing Attitudes in Germany 2016].* Gießen: Psychosozial-Verlag.

[B21] Decker O. Kiess J. Schuler J. Handke B. Brähler E. (2018). “Die leipziger autoritarismus-studie 2018: methode, ergebnisse und langzeitverlauf [The Leipzig study of authoritarianism 2018: methods, results and long term developments],” in *Flucht ins Autoritäre. Rechtsextreme Dynamiken in der Mitte der Gesellschaft*, eds Decker O. Brähler E. (Gießen: Psychosozial-Verlag), 65–116.

[B22] Duckitt J. (2015). “Authoritarian personality,” in *International Encyclopedia of the Social & Behavioral Sciences*, 2nd Edn, ed. Wright J. D. (Amsterdam: Elsevier), 255–261. 10.1016/B978-0-08-097086-8.24042-7

[B23] Duckitt J. Bizumic B. (2013). Multidimensionality of right-wing authoritarian attitudes: authoritarianism-conservatism-traditionalism. *Political Psychol.* 34 841–862. 10.1111/pops.12022

[B24] Duckitt J. Bizumic B. Krauss S. W. Heled E. (2010). A tripartite approach to right-wing authoritarianism: the authoritarianism-conservatism-traditionalism model. *Political Psychol.* 31 685–715. 10.1111/j.1467-9221.2010.00781.x

[B25] Duckitt J. Sibley C. G. (2007). Right wing authoritarianism, social dominance orientation and the dimensions of generalized prejudice. *Eur. J. Pers.* 21 113–130. 10.1002/per.614

[B26] Duckitt J. Sibley C. G. (2009). A dual-process motivational model of ideology, politics, and prejudice. *Psychol. Inquiry* 20 98–109. 10.1080/10478400903028540

[B27] Dunwoody P. T. Plane D. L. (2019). The influence of authoritarianism and outgroup threat on political affiliations and support for antidemocratic policies. *Peace Conflict J. Peace Psychol.* 25 198–210. 10.1037/pac0000397

[B28] Ekehammar B. Akrami N. Gylje M. Zakrisson I. (2004). What matters most to prejudice: big five personality, social dominance orientation, or right-wing authoritarianism? *Eur. J. Pers.* 18 463–482. 10.1002/per.526

[B29] Federal Statistical Office of Germany (2019). *Bevölkerung* [Population]. Available online at: https://www.destatis.de/DE/Themen/Gesellschaft-Umwelt/Bevoelkerung/_inhalt.html (accessed November 29, 2019).

[B30] Funke F. (2005). The dimensionality of right-wing authoritarianism: lessons from the dilemma between theory and measurement. *Political Psychol.* 26 195–218. 10.1111/j.1467-9221.2005.00415.x

[B31] Gregorich S. E. (2006). Do self-report instruments allow meaningful comparisons across diverse population groups? Testing measurement invariance using the confirmatory factor analysis framework. *Medical Care* 44 78–94. 10.1097/01.mlr.0000245454.12228.8f PMC1808350 17060839

[B32] Heitmeyer W. (2012). “Gruppenbezogene menschenfeindlichkeit (GMF) in einem entsicherten jahrzehnt [Group-related hostility in an unsafe decade],” in *Deutsche Zustände 10*, ed. Heitmeyer W. (Berlin: Suhrkamp), 15–41. 10.2307/j.ctvddzr0c.4

[B33] Horn J. L. (1965). A rationale and test for the number of factors in factor analysis. *Psychometrika* 30 179–185. 10.1007/bf02289447 14306381

[B34] Jost J. T. Glaser J. Kruglanski A. W. Sulloway F. J. (2003). Political conservatism as motivated social cognition. *Psychol. Bull.* 129 339–375. 10.1037/0033-2909.129.3.339 12784934

[B35] Lee K. Ashton M. C. Griep Y. Edmonds M. (2018). Personality, religion, and politics: An investigation in 33 countries. *Eur. J. Pers.* 32 100–115. 10.1002/per.2142

[B36] MacWilliams M. C. (2016). *“Measuring Authoritarianism”. The Rise of Trump.* Available online at: https://acpress.amherst.edu/books/riseoftrump/chapter/measuring-authoritarianism/ (accessed November 29, 2019).

[B37] Marsh H. W. Guo J. Parker P. D. Nagengast B. Asparouhov T. Muthén B. O. (2018). What to do when scalar invariance fails: the extended alignment method for multi-group factor analysis comparison of latent means across many groups. *Psychol. Methods* 23 524–545. 10.1037/met0000113 28080078

[B38] McDonald R. (1999). *Test Theory: a Unified Treatment.* Mahwah, NJ: Lawrence Erlbaum.

[B39] Meredith W. (1993). Measurement invariance, factor analysis and factorial invariance. *Psychometrika* 58 525–543. 10.1007/BF02294825

[B40] Milfont T. L. Fischer R. (2010). Testing measurement invariance across groups: applications in cross-cultural research. *Int. J. Psychol. Res.* 3 111–121. 10.21500/20112084.857

[B41] Moosbrugger H. Kelava A. (2012). *Testtheorie und Fragebogenkonstruktion. [Test Theory and Construction of Questionnaires.].* Berlin: Springer Verlag.

[B42] O’Connor B. P. (2000). SPSS and SAS programs for determining the number of components using parallel analysis and Velicer’s MAP test. *Behav. Res. Methods Instruments Comp.* 32 396–402. 10.3758/BF03200807 11029811

[B43] Oesterreich D. (2005). Flight into security: a new approach and measure of the authoritarian personality. *Political Psychol.* 26 275–297. 10.1111/j.1467-9221.2005.00418.x

[B44] Patil V. H. Singh S. N. Mishra S. Donavan D. T. (2017). *Parallel Analysis Engine to Aid in Determining Number of Factors to Retain Using R [Computer Software].* Available online at: https://analytics.gonzaga.edu/parallelengine/ (accessed November 29, 2019).

[B45] Pituch K. A. Stevens J. P. (2016). *Applied Multivariate Statistics for the Social Sciences: Analyses with SAS and IBM’s SPSS.* Abingdon: Routledge.

[B46] Podsakoff P. M. MacKenzie S. B. Lee J.-Y. Podsakoff N. P. (2003). Common method biases in behavioral research: a critical review of the literature and recommended remedies. *J. Appl. Psychol.* 88 879–903. 10.1037/0021-9010.88.5.879 14516251

[B47] Putnick D. L. Bornstein M. H. (2016). Measurement invariance conventions and reporting: the state of the art and future directions for psychological research. *Dev. Rev.* 41 71–90. 10.1016/j.dr.2016.06.004 27942093 PMC5145197

[B48] Ray J. J. (1990). Acquiescence and problems with forced-choice scales. *J. Soc. Psychol.* 130 397–399. 10.1080/00224545.1990.9924595

[B49] Rippl S. (2002). Bildung und Fremdenfeindlichkeit. Die Rolle schulischer und familialer Sozialisation zur Erklärung von Bildungsunterschieden im Ausmaß von fremdenfeindlichen Einstellungen [Education and xenophobia. The role of academic and familial socialization in explaining the differences in education regarding the extent of xenophobic attitudes]. *Kölner Zeitschr. Soziol. Sozialpsychol.* 54 135–146. 10.1007/s11577-002-0006-0

[B50] Rokeach M. (1967). Authoritarianism scales and response bias: comment on Peabody’s paper. *Psychol. Bull.* 67 349–355. 10.1037/h0024534 6047175

[B51] Rosseel Y. (2012). lavaan: an R package for structural equation modeling. *J. Stat. Softw.* 48 1–36. 10.18637/jss.v048.i02

[B52] Satorra A. Bentler P. M. (2001). A scaled difference chi-square test statistic for moment structure analysis. *Psychometrika* 66 507–514. 10.1007/BF02296192 PMC2905175 20640194

[B53] Saunders B. A. Ngo J. (2017). “The right-wing authoritarianism scale,” in *Encyclopedia of Personality and Individual Differences*, eds Zeigler-Hill V. Shackelford T. K. (Basel: Springer International Publishing AG).

[B54] Schmidt P. Stephan K. Herrmann A. (1995). “Entwicklung einer Kurzskala zur messung von autoritarismus [Development of a short scale to measure authoritarianism],” in *Autoritarismus und Gesellschaft: Trendanalysen und Vergleichende Jugenduntersuchungen von 1945–1993*, eds Lederer G. Schmidt P. (Opladen: Leske & Budrich), 221–227. 10.1007/978-3-322-91401-9_10

[B55] semTools Contributors (2016). *SemTools: Useful Tools for Structural Equation Modeling* (R package version 0.4-14). Available online at: https://CRAN.R-project.org/package=semTools (accessed November 29, 2019).

[B56] Steiger J. H. (1989). *EzPATH: Causal Modeling.* Evanston, IL: SYSTAT.

[B57] Trizano-Hermosilla I. Alvarado J. M. (2016). Best alternatives to Cronbach’s Alpha reliability in realistic conditions: congeneric and asymmetrical measurements. *Front. Psychol.* 7:796. 10.3389/fpsyg.2016.00769 27303333 PMC4880791

[B58] Zick A. Küpper B. Hövermann A. (2011). *Die Abwertung der Anderen. Eine Europäische Zustandsbeschreibung zu Intoleranz, Vorurteilen und Diskriminierung [Devaluation of Others. A Description of the European State regarding Intolerance, Prejudice and Discrimination].* Berlin: FES.

[B59] Ziegler M. Kemper C. Kruyen P. (2014). Short scales: five misunderstandings and ways to overcome. *J. Individ. Diff.* 34 185–189. 10.1027/1614-0001/a000148

